# The Book World of Medicine and Science

**Published:** 1906-09-08

**Authors:** 


### The Book World of Medicine and Science.

The Science and Art of Prescribing. By E. H. Colbeck, B.A., M.D.(Cantab.), F.R.C.P.(Lond.), D.P.H. (Cantab.), and Arnold Chaplin, B.A., M.D.(Cantab.), F.R.C.P.(Lond.). Second Edition, revised and enlarged. (London : Henry Kimpton, 13 Furnival Street, Holborn, E.C. 1906. Price 3s. 6d.)

This book ought to be a favourite with the general practitioner who has much dispensing to do. It is a short and reliable guide to the art of prescribing, and endeavours to clarify the difficulties of dispensing to the student who is afforded so little opportunity at the present day of mastering this essential and indispensable acquirement. The use of ready-made drugs will not make up for neglect of the art of prescribing. This little volume gives a good account of the prescription, of incompatibility in all its forms, and the methods of administration of drugs. No one needs to think himself perfect in these subjects, or that excellent hints are not obtainable from this work. There is a useful table giving the dose and correction of taste of drugs which should be valuable both to prescribers and partakers, as also one of drugs suitable for use as gargles, sprays, and pigments with the requisite vehicle and quantities. The best part is that which deals with the application of methods of prescribing arranged according to the various systems, the classification of diseases having been adopted for the purpose of illustrating the various methods of administering drugs. Thus, under the alimentary system, the diseases of the mouth, pharynx, stomach, liver, and intestines are comprised, and various washes, mixtures, gargles, pigments, injections, powders, pills, and draughts are illustrated by suitable prescriptions. The vascular system is similarly subdivided and dealt with, and many useful hints as to the best treatment of the conditions are supplied. So with the other systems of the body and with those diseases which are described as "obscure causation." The work has an important appendix dealing with the approximate ingredients of various patent and proprietary preparations—a very necessary addition in view of the pernicious practice of self-drugging.

The Morphology of Normal and Pathological Blood. By George A. Buckmaster, D.M.(Oxon.). (London : John Murray. 10s. 6d. net.)

This book is composed of a series of lectures delivered at University College. The scientific study of the blood may almost be said to be a study which was not in existence comparatively few years ago. It is at least scarcely older than that of bacteriology and these two branches of medical science have important bearings one upon the other. These lectures give a valuable summary of important and extensive knowledge that has already been acquired. Amongst the most interesting chapters is that dealing with the tests for blood. The subject is medico-legal rather than purely medical, but is none the less important. Amongst the numerous constituents of normal sera are the preciptins, and study of these preciptins has shown what has hitherto so puzzled medical jurists, that it is possible to distinguish human from other forms of mammalian blood, and also to distinguish the blood of one animal from that of another. This test has already been put on trial successfully in cases of murder in Germany and Austria, and in one remarkable case the blood expert returned in his report that the blood on the clothes sent for examination was a mixture of sheep's and human blood. External evidence proved this. It was found that the accused had been slaughtering sheep two weeks before he committed the murder. The book contains photographs of blood cells, coloured illustrations, and diagrams to illustrate some of the complicated problems that have presented themselves to students of the physiology and diseases of blood.

The Elements of the Practice of Comparative Medicine, together with Records of some Cases. By Frank Townend Barton, M.R.C.V.S., and George Gresswell, M.A.(Oxon.), L.R.C.P., L.R.C.S.(Edin.) (London : Everett and Company, 42 Essex Street, Strand, W.C.)

This is the first book which has appeared devoted to what is more or less a new departure in scientific nomenclature "Comparative Medicine," the relation of the diseases of the lower animals to man. The work gives an outline of the principal clinical features of some of the diseases. The subjects dealt with are glanders, anthrax, tuberculosis, tetanus, actinomycosis, and rabies. These diseases most practitioners would recognise in man, but might fail if asked to detect them in the horse, sheep, pig, dog, or ox. Similarly with foot and mouth disease, which is communicable to man and with the tick disease, the itch, trichiniasis, and psorospermiasis. There is, further, the group of malarial and other tropical animal-borne diseases about which modern research has learned so much. The authors also note hydatid disease and disorders due to the growth of fungi on the skin. It is true the work is by no means exhaustive, but it is suggestive, instructive, well and simply written, and above all practical and of the greatest service to all who have dealings with the lower animals.

The World's Anatomists. By G. W. H. Kemper, M.D. (Philadelphia : P. Blakiston, Son and Company.)

Our cousins in America seem to be more interested in the history of medicine than ourselves. There have been great men in the past in many branches of medicine, but perhaps the work of the anatomists from its very nature has been the least chimerical, and the names of various structures in our body remind us of workers who lived many centuries ago. This little book arranges brief notes, in alphabetical order, of great anatomists from the time of Theophilus 335 B.C. to the present day, with here and there an artistic illustration reproduced from an old print or recent photograph. The author in his preface describes how fascinating has been the study of the lives of these men, many of whom "toiled and toiled"—worked like Scarpa until the age of "three-score years and ten," when blindness overtook him, or maintained their enthusiasm like Rugsh, who was still busy at the age of ninety-three. Information like this brings home to our minds the fact that the multitudinous details of description which comprise the science of human anatomy were not gathered in a day and that we have to thank men of long ago for what seems now simple and plain.

Food in Health and Disease. By H. Drinkwater. (London : J. M. Dent and Company. 1s.)

A preface to this little book by Dr. Bradshawe of Liverpool describes the author as a practitioner of medicine engaged in "arduous practice who has found time to keep himself abreast of the progress of medical sciences." The energy of character which enables a medical man to write in the midst of the unsettling and irregular duties of general practice is a quality to be admired. Food occupies an important place when a man's thought is directed towards himself and questions concerning it are amongst the most common that are presented to a medical man by his patients. In this book will be found essential facts concerning the multitudinous articles of diet of value in health and disease.

